# Gastrointestinal Dysfunctions Are Associated with *IL-10* Variants in Parkinson's Disease

**DOI:** 10.1155/2018/5908359

**Published:** 2018-12-05

**Authors:** Li Shu, Dongxiao Liang, Hongxu Pan, Qian Xu, Xinxiang Yan, Beisha Tang, Qiying Sun

**Affiliations:** ^1^Department of Neurology, Xiangya Hospital, Central South University, Changsha, Hunan 410008, China; ^2^National Clinical Research Center for Geriatric Disorders, Changsha, Hunan 410078, China; ^3^Key Laboratory of Hunan Province in Neurodegenerative Disorders, Central South University, Changsha, Hunan 410008, China; ^4^Department of Geriatrics, Xiangya Hospital, Central South University, Changsha, Hunan 410008, China; ^5^Center for Medical Genetics, Central South University, Changsha, Hunan 410008, China; ^6^Parkinson's Disease Center of Beijing Institute for Brain Disorders, Beijing 100069, China

## Abstract

Inflammation has been demonstrated to be involved in Parkinson's disease (PD) pathogenesis. There were evidences that the disturbance of the protective function of *IL-10* gene contributed to PD. In our study, haplotype analyses were conducted of *IL-10* rs1800871 and rs1800872 on 371 PD patients. Because the two SNPs exposed significant linkage disequilibrium demonstrated by Haploview software, we included 177 carriers of both rs1800871 and rs1800872 and 190 noncarriers in clinical phenotype analyses. As to nonmotor symptoms, the score of the gastrointestinal dysfunction domain in Nonmotor Symptom Scale (NMSS) was lower in the carrier group of both SNPs than in the noncarrier group in PD patients (SC: −0.198, *p* : 023). Other nonmotor symptoms reflected by relevant rating scales showed negative results. As to comorbidity, no significant statistical significance was observed between the two SNPs and Charlson Comorbidity Index (CCI). In conclusion, we found less severe gastrointestinal dysfunctions of both *IL-10* rs1800871 and rs1800872 carriers than noncarriers in PD.

## 1. Introduction

Parkinson's disease (PD) is a common neurodegenerative disease. The pathogenic hallmarks are dopamine neuron death and Lewy body aggregation in substantia nigra (SNc) in PD. The etiology of PD remains elusive. The genetic factor, environmental factor accompanied with aging contributes to PD pathogenesis [1–4]. Neuroinflammatory process may be one of the factors contributing to pathogenesis of PD. Evidences had proved that the neuroinflammation took part in dopamine neuron death in SNc. Microglia were acted and they secreted cytokines, chemokines, oxygen/nitrogen species, etc., which were detrimental to dopamine neurons [5].

Inflammatory cytokines such as tumor necrosis factors (TNFs) and interleukins (ILs) had been proved to exert effects on PD pathogenesis in the brain and in the peripheral. Postmortem analyses of PD patients' brains and cerebrospinal fluids showed proinflammatory cytokines accumulations [6]. Significant elevations of levels of proinflammatory cytokines IL-6, TNF, IL-1*β*, C-reactive protein (CRP), and IL-2 were found in PD patients compared with controls in the peripheral blood [7]. Mice deficient in the inflammatory genes were observed to exert a protective role against toxicity of dopamine neurons [8].

IL-10 encoded by *IL-10* gene is an anti-inflammatory factor, which is produced by lymphocytes and mononuclear phagocytes including microglia. There was evidence that the level of IL-10 was higher in the PD group than in the control group in serum [9]. In the IL-10-based therapies in animal models of PD, IL-10 was also protective against LPS-induced loss of dopaminergic cells. The injection of IL-10 can be protective by increasing striatal tyrosine hydroxylase in the 1-methyl-4-phenyl-1,2,3,6-tetrahydropyridine (MPTP) model of PD [10]. Rs1800872 (−819 T/C) and rs1800871 (−592 A/C) SNPs were common variants in the promoter region of the gene and had a functional effect on *IL-10* gene transcription [11]. Multiple studies focused on the relationship between the two variants and neurological diseases such as Alzheimer's disease [12], schizophrenia [13], especially PD [14–16], and validated their role on the diseases.

There were few studies talking about *IL-10*-related clinical features in PD and the sample sizes were small. *IL-10* rs1800872 was reported to be associated with early onset and female PD [16]. No relationship was found between *IL-10* variants and nonmotor symptoms, such as cognitive dysfunction and depression [17, 18]. In our analysis, we included a large sample of Chinese populations to do an association analysis on the relationship between rs1800871 and rs1800872 in *IL-10* and the clinical features of PD.

## 2. Methods

### 2.1. Participants

All the PD patients were recruited from the Xiangya Hospital of Central South University randomly, and everyone agreed to participate in the study. Patients were diagnosed using the United Kingdom Parkinson's Disease Brain Bank by movement disorder specialists [19]. A total of 371 patients were recruited and the genotyping was done by Liu Z et al [17]. In detail, there were 177 rs1800871 carriers (CC 42 + AC 135), 194 rs1800871 noncarriers (AA 194), 181 rs1800872 carriers (CC 47 + TC 134), and 190 rs1800872 noncarriers (TT 190) included in our analyses. The study was conducted according to Helsinki and was approved by the Ethics Committee of Xiangya Hospital. All the patients provided informed consent in our study.

### 2.2. Clinical Assessment

Unified Parkinson's Disease Rating Scale part I (UPDRS I) was used to assess mentation, behavior, and mood, and UPDRS II was performed to evaluate the activities of daily life. Motor symptoms were assessed by UPDRS III. The Hoehn and Yahr scale (H-Y) was performed to assess the disease stage [20]. We also calculated the levodopa equivalent daily dose (LEDD). The wearing-off (WO) phenomenon was the shortening therapeutic effect of levodopa and it was assessed by a 9-item Wearing-off Questionnaire (WOQ-9) [21, 22]. The phenomenon of freezing of gait (FOG) was characterized by brief episodes of inability to step or by small shuffling steps, and it was evaluated by the New Freezing of Gait Questionnaire (NFOG-Q) [23, 24].

Cognitive function was evaluated by the Mini-Mental State Examination (MMSE). The score lower than 26 points indicated a cognitive impairment [25]. Parkinson's Disease Sleep Scale (PDSS) was used to assess sleep disturbances. The REM sleep behavior disorder (RBD) was evaluated by REM Sleep Behavior Disorder Questionnaire-Hong Kong (RBDQ-HK) and RBD as defined with a recommended cutoff value of 18 [26]. The Epworth Sleepiness Scale (ESS) was used to assess the excessive daytime sleepiness (EDS). A score of 10 or higher may indicate that the person had excessive daytime sleepiness [27]. Olfactory dysfunction was assessed by the Hyposmia Rating Scale (HRS). 22.5 was the cutoff point of HRS [28]. We used the domains of the Nonmotor Symptoms Scale (NMSS) to assess a series of nonmotor symptoms including cardiovascular domain, sleep/fatigue domain, mood/apathy domain, perception/hallucinations domain, attention/memory domain, gastrointestinal domain, urinary domain, and sexual dysfunction domain [29, 30].

39-item Parkinson's Disease Questionnaire (PDQ-39) was used to assess the quality of life [31]. The Charlson Comorbidity Index (CCI) score was used to assess the comorbidity conditions [32]. We can predict the burden of multiple chronic illnesses [33].

### 2.3. Statistical Analysis

To compare the demographic and the clinical features among different genotypes carriers, summary statistics were divided into numerical variables and categorical variables. SPSS software (version 22.0; SPSS Inc) was used to perform statistical analyses.

We used mean and standard deviation to present numerical variables. The linear regression model was used to evaluate the association between numerical variables of IL-10 variants and clinical features of PD after adjusting by age and gender. The absolute value of standardized coefficient (SC) was to reflect the effect of the variation on clinical symptoms. Statistical significance was set at *p* < 0.05.

Categorical variables were presented as number of carriers or noncarriers and its relevant frequency separately. The binary logistic regression model was used to test the relation of categorical variables and genotypes after adjusting by age and gender. Odds ratio (OR) and 95% confidence interval (CI) were estimated. The value of OR > 1 was considered as a risk factor. Statistical significance was set at *p* < 0.05.

Haploview software was used for haplotype analysis. The color schemes of linkage disequilibrium (LD) plot in the software represent LD relationships. The color is defined according to the value of coefficient D′ and logarithm of odds (LOD). If |D'| < 1 and LOD < 2, the color is white; if |D'| < 1 and LOD ≥ 2, the color is pink/red [34].

The expression quantitative trait loci (eQTL) of the two variants were found by searching the Braineac database.

## 3. Results

### 3.1. Haplotype Analysis

**Figure 1:**
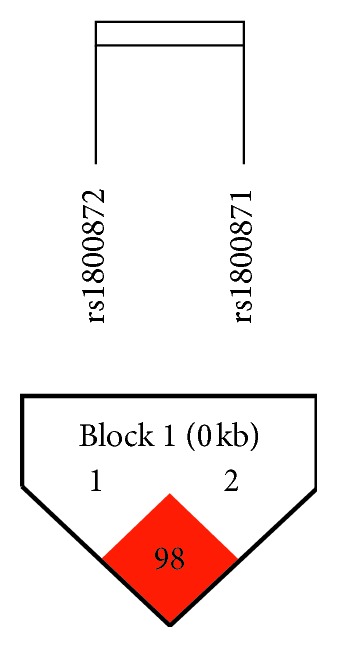
The haplotype analysis of rs1800871 and rs1800872 by Haploview software.

The haplotype analysis by Haploview software is demonstrated in Figure 1. Red of the block meant that the two SNPs exposed significant LD (D': 0.987, LOD: 130.95), indicating that the two variants exposed significant linkage disequilibrium.

### 3.2. Clinical Features of PD Patients Carrying Both rs1800871 and rs1800872

We included 177 carriers of both rs1800871 and rs1800872 and 190 noncarriers in clinical phenotype analyses. The mean age of the carrier group and noncarrier group of both polymorphisms were 62.64 ± 10.46 years and 63.23 ± 9.49 years, respectively.

There was no statistical significance between the variant and UPDRS I, UPDRS II, and UPDRS III scores, between the carrier group and noncarrier group (SC: −0.002, *p* : 0.977, SC: −0.054, *p* : 0.319, SC: −0.075, *p* : 0.157). The H-Y score in the carriers were similar to noncarriers (SC: −0.070, *p* : 0.211). No significant statistical differences were found between the variant and LEDD (SC: 0.201, *p* : 0.070), the frequency of WO phenomenon assessed by WOQ-9 (OR: 0.692, 95%CI: 0.365–1.311, *p* : 0.258) and the frequency of FOG assessed by NFOG-Q (OR: 0.811, 95%CI: 0.348–1.887, *p* : 0.626) (Tables 1 and 2).

The frequency of cognitive impairments assessed by MMSE in carriers was similar to noncarriers (OR: 1.263, 95%CI: 0.644–2.478, *p* : 0.497). The frequency of olfactory dysfunction assessed by HRS did not present with statistical differences between two groups (OR: 0.843, 95%CI: 0.418–1.700, *p* : 0.633). In addition, we also found that there was no statistical significance between the variant and depression assessed by HAMD (OR: 0.938, 95%CI: 0.496–1.774, *p* : 0.844) (Tables 1 and 2).

For sleep disturbances, the PDSS score in the carrier group was similar to the noncarrier group (SC: −0.039, *p* : 0.657). The frequency of sleep disturbances assessed by the other 2 scales (RBD-HK, ESS) showed no statistically significant differences between the carriers and noncarriers in PD (OR: 1.122, 95%CI: 0.553–2.277, *p* : 0.749; OR: 0.686, 95%CI: 0.329–1.430, *p* : 0.314) (Tables 1 and 2).

The score of the gastrointestinal dysfunction domain in NMSS was lower in carriers than in noncarriers (SC: –0.198, *p* : 0.023). The scores of the cardiovascular domain, sleep/fatigue domain, mood/apathy domain, perception/hallucinations domain, attention/memory domain, urinary domain, and sexual dysfunction domain of NMSS in the carriers were similar to those of noncarriers (SC: 0.062, *p* : 0.492; SC: 0.087, *p* : 0.334; SC: −0.016, *p* : 0.862; SC: 0.020, *p* : 0.818; SC: −0.105, *p* : 0.235; SC: −0.040, *p* : 0.649; SC: 0.057, *p* : 0.524) (Tables 1 and 2).

For the quality of life, there was no statistical significance between the variant and PDQ-39 score (SC: 0.079, *p* : 0.376). For comorbidity conditions, the CCI score of carriers was similar to that of noncarriers (SC: −0.059, *p* : 0.259) (Tables 1 and 2).

### 3.3. eQTL Analysis

In the Braineac database, we found that the eQTLs of rs1800871 were inflammatory-related genes such as *CR1L*, *IL-20* while those of rs1800872 were *CR1L*, *IL-20*, *IL-10* (Supplementary Tables 1 and 2).

## 4. Discussion

Our analysis is a comprehensive analysis on the clinical features of *IL-10* rs1800872 and rs1800871 in PD. Data of motor symptoms, nonmotor symptoms, and comorbidities were all collected in a large sample of PD patients of Chinese origin. In our analysis, less severe gastrointestinal dysfunctions were observed in the carrier group of both the rs1800871 and rs1800872 than in the noncarrier group in PD patients.

Gastrointestinal dysfunction is a common nonmotor symptom in PD which can both precede the classic motor symptoms up to ten years and be present in over 80% symptomatic PD patients [35, 36]. Pathological findings had identified *α*-synuclein in PD patients' intestinal biopsies which led the subsequent degeneration of enteric nervous system [37]. Additionally, researchers had found that, in a PD mouse model, there was a decrease in colonic mobility and an increase in constipation [38]. The peripheral inflammation such as intestinal inflammation can cause such abnormal protein mobilizing between the gut and the brain and cause dopaminergic neurons to degeneration [39, 40].

Multiple proinflammatory immune factors were proved to be associated with gastrointestinal symptoms in PD. In large bowels in PD patients, there were increased levels of cytokines such as tumor necrosis factor (TNF), IL-1b, IL-6, and interferon-c (IFN-c) [38]. Increased levels of IL-1, IL-8, and C-reactive protein were also observed in the stool of PD [37]. Our analysis widened the spectrum of immune factors related to gastrointestinal symptoms in PD by proving the possible role of anti-inflammatory factor IL-10 in PD clinical phenotypes. The variants in the gene may possibly affect the function of gastrointestinal of PD by affecting the expressions of *IL-10* or other inflammatory factor genes and thus modulating the aggregations of *α*-synuclein in the gastrointestinal tract system.

Identifying *IL-10*-related clinical phenotypes will contribute to the genetic counseling of PD. The *IL-10-*related gastrointestinal dysfunctions in PD might provide a clue for symptomatic treatment for PD. *IL-10*-based gene therapies in various neuroimmune diseases have been proved to be effective at treating both the symptoms and pathology [10]. In order to further explore the role of *IL-10* in PD, large sample, multicenter, randomized controlled cohorts with comprehensive genetic and clinical analyses were needed to evaluate the therapeutic potential of *IL-10* in neuroimmune and neurodegenerative disease.

Limitations still existed in our study. Although with large samples, our study was still a cross-sectional study. Longitudinal, multicentred studies were needed to reach a more convincing result. Additionally, for lack of enough clinical data, other clinical features such as olfactory dysfunction and fatigue except for those in our study were not analyzed. Moreover, the interactions among different variants from various genes which may affect the clinical features of PD were not analyzed in our study.

## 5. Conclusion

In conclusion, there were less severe gastrointestinal dysfunctions of both *IL-10* rs1800871 and rs1800872 carriers than noncarriers in PD.

## Figures and Tables

**Table 1:** Demographic data and clinical characteristics of PD patients in both *IL-10* rs1800871 and rs1800872 carriers and noncarriers (numerical variables).

	Carriers		Noncarriers		*p*	SC
	*N*	*X* ± *S*	*N*	*X* ± *S*		
Age	177	62.64 ± 10.46	190	63.23 ± 9.49	NA	NA
UPDRS I	165	2.30 ± 1.97	174	2.32 ± 2.37	0.977	−0.002
UPDRS II	165	12.88 ± 6.49	174	13.78 ± 8.08	0.319	−0.054
UPDRS III	172	26.76 ± 14.70	178	29.26 ± 16.01	0.157	−0.075
H-Y	156	2.27 ± 0.93	161	2.40 ± 0.96	0.211	−0.070
LEDD	166	556.08 ± 281.23	178	517.52 ± 314.51	0.201	0.070
PDSS	67	106.39 ± 23.33	70	107.13 ± 27.26	0.657	−0.039
NMSS-D1	64	0.97 ± 2.03	68	0.79 ± 1.78	0.492	0.062
NMSS-D2	64	10.88 ± 6.67	68	9.79 ± 6.95	0.334	0.087
NMSS-D3	64	7.97 ± 9.78	68	7.71 ± 9.52	0.862	−0.016
NMSS-D4	64	1.61 ± 2.58	68	1.69 ± 3.23	0.818	0.020
NMSS-D5	64	3.25 ± 3.82	68	4.44 ± 4.68	0.235	−0.105
NMSS-D6	64	4.25 ± 4.58	68	6.07 ± 4.76	**0.023**	−**0.198**
NMSS-D7	64	6.55 ± 5.29	68	7.18 ± 6.02	0.649	−0.040
NMSS-D8	64	0.92 ± 3.05	68	0.53 ± 2.43	0.524	0.057
PDQ-39	64	36.33 ± 25.68	70	32.91 ± 23.39	0.376	0.079
CCI	177	0.12 ± 0.40	190	0.17 ± 0.50	0.259	−0.059

Abbreviations: UPDRS I, II, III, Unified Parkinson's Disease Rating Scale part I, II, III; H-Y, Hoehn and Yahr Scale; LEDD, Levodopa equivalent daily dose; PDSS, Parkinson's Disease Sleep Scale; NMSS, Nonmotor Symptoms Scale; NMSS-D1, NMSS-cardiovascular; NMSS-D2, NMSS-sleep/fatigue; NMSS-D3, NMSS-mood/apathy; NMSS-D4, NMSS-perception/hallucinations; NMSS-D5, NMSS-attention/memory; NMSS-D6, NMSS-gastrointestinal; NMSS-D7, NMSS-urinary; NMSS-D8, NMSS-sexual dysfunction; PDQ-39, the 39-item Parkinson's Disease Questionnaire; CCI, Charlson Comorbidity Index. N represented the total number of carriers and noncarriers of both IL-10 rs1800871 and rs1800872 in PD patients. Numerical variable was presented as mean and standard deviation (*X* ± *S*). *P* values <0.05 were considered of statistical significance and was shown in boldface text. SC, standardized coefficient; NA, not available.

**Table 2:** Demographic data and clinical characteristics of PD patients in both *IL-10* rs1800871 and rs1800872 carriers and noncarriers (categorical variables).

	Carriers		Noncarriers		*p*	OR	95% CI
	*N*	*N* ^*∗*^ (*F*)	*N*	*N* ^*∗*^ (*F*)			
Gender, male	177	91(51.4%)	190	97(51.1%)	NA	NA	NA
WO	159	19(11.9%)	159	26(16.4%)	0.258	0.692	0.365–1.311
FOG	46	14(30.4%)	56	20(35.7%)	0.626	0.811	0.348–1.887
Cognitive impairment	96	26(27.1%)	96	25(26.0%)	0.497	1.263	0.644–2.478
Olfactory dysfunction	63	29(46.0%)	68	35(51.5%)	0.633	0.843	0.418–1.700
Depression	84	32(38.1%)	82	32(39.0%)	0.844	0.938	0.496–1.774
RBD	63	27(42.9%)	70	30(42.9%)	0.749	1.122	0.553–2.277
EDS	65	20(30.8%)	72	31(43.1%)	0.314	0.686	0.329–1.430

Abbreviations: WO, wearing-off phenomenon which was evaluated by 9-item Wearing-off Questionnaire (WOQ-9); FOG, freezing of gait which was assessed by the New Freezing of Gait Questionnaire (NFOG-Q); cognitive impairment which was assessed by mini mental state examination (MMSE); Olfactory dysfunction which was assessed by the Hyposmia Rating Scale (HRS); depression which was assessed by the Hamilton Depression Scale (HAMD); RBD, REM sleep behavior disorder which was assessed by REM Sleep Behavior Disorder Questionnaire-Hong Kong (RBDQ-HK); EDS, excessive daytime sleepiness which was assessed by the Epworth Sleepiness Scale (ESS). N represented the total number of carriers and noncarriers of both IL-10 rs1800871 and rs1800872 in PD patients. Categorical variable was presented as number of carriers or noncarriers and its relevant frequency separately [N^*∗*^ (F)]. *P* value <0.05 was considered of statistical significance. OR, odds ratio; CI, confidence interval; NA, not available.

## Data Availability

The data used to support the findings of this study are included within the article and the supplementary file.

## Supplementary Materials

Supplementary Materials Tables S1 and S2 show the results of eQTLs from Braineac database.
