# Supplementary material for: Gastrointestinal Dysfunctions Are Associated with IL-10 Variants in Parkinson's Disease
Source: Parkinsons Dis. 2018 Dec 5;2018:5908359. doi: 10.1155/2018/5908359 (PMC6304865; doi:10.1155/2018/5908359)
Supplement: Supplementary Materials — Tables S1 and S2 show the results of eQTLs from Braineac database. [file 5908359.f1.pdf]

Supplementary Table 1. Results of *IL-10* rs1800871 eQTLs from Braineac database.

**geneSymbol**	**marker**	**rsid**	**exprID**	**chr**	**start**	**stop**	**aveALL**	**CRBL**	**FCTX**	**HIPP**	**MEDU**	**OCTX**	**PUTM**	**SNIG**	**TCTX**	**THAL**	**WHMT**
IKBKE,C1orf147	chr1:206946634	rs1800871	2376833	chr1	206643758	206671379	8.80E-01	6.20E-01	8.20E-01	7.00E-02	3.50E-01	3.60E-01	9.40E-01	**2.20E-04**	9.50E-01	5.20E-02	9.70E-01
C4BPA	chr1:206946634	rs1800871	2377185	chr1	207277590	207332055	4.60E-01	**7.60E-04**	9.30E-01	6.50E-01	4.70E-01	2.90E-01	3.10E-01	8.80E-01	9.30E-01	7.90E-01	4.30E-01
FAIM3	chr1:206946634	rs1800871	2452987	chr1	207076630	207101034	**1.30E-02**	3.20E-01	2.20E-01	3.00E-01	5.00E-02	5.60E-01	6.80E-01	3.10E-01	5.60E-01	5.40E-01	**9.70E-04**
LGTN	chr1:206946634	rs1800871	2452902	chr1	206744640	206785896	5.00E-01	7.50E-01	2.30E-01	6.70E-01	8.20E-01	4.00E-01	4.80E-01	**1.20E-03**	7.20E-01	9.50E-01	**1.20E-02**
PIGR	chr1:206946634	rs1800871	2453011	chr1	207101875	207119807	1.90E-01	8.30E-01	9.70E-01	**2.20E-02**	3.60E-01	5.40E-01	4.50E-01	**1.50E-03**	9.80E-01	4.80E-01	**9.30E-03**
CD46,CR1L	chr1:206946634	rs1800871	2377507	chr1	207818478	207983220	1.60E-01	7.70E-01	5.60E-01	2.60E-01	8.10E-01	2.30E-01	9.60E-02	1.90E-01	5.50E-01	**1.70E-03**	4.40E-01
PIGR	chr1:206946634	rs1800871	2453018	chr1	207101875	207119807	2.90E-01	9.20E-01	8.40E-01	3.60E-01	**1.90E-03**	2.10E-01	6.90E-02	2.80E-01	2.40E-01	7.60E-01	1.20E-01
IL20	chr1:206946634	rs1800871	2377026	chr1	207038709	207042565	**4.00E-03**	3.00E-01	**2.60E-03**	8.80E-02	5.80E-01	9.60E-02	1.90E-01	3.10E-01	5.60E-01	7.90E-01	8.80E-02
CR1,CR1L	chr1:206946634	rs1800871	2377424	chr1	207669246	207815110	3.60E-01	3.10E-01	1.30E-01	4.40E-01	**2.70E-03**	7.10E-01	2.70E-01	**4.40E-02**	2.40E-01	4.70E-01	8.60E-01
C4BPA	chr1:206946634	rs1800871	2377169	chr1	207277590	207332055	7.50E-01	2.70E-01	7.20E-01	3.40E-01	2.20E-01	6.70E-01	3.50E-01	**3.30E-03**	8.00E-02	4.00E-01	2.10E-01

Abbreviations: Ave All, average across all ten regions; CRBL, cerebellum; FCTX, frontal cortex; HIPP, hippocampus; MEDU, medulla; OCTX, occipital cortex; PUTM, putamen; SNIG, substantia nigra; TCTX, temporal cortex; THAL, thalamus; WHMT, white matter. The bold text represents statistically significant results.

Supplementary Table 2. Results of *IL-10* rs1800872 eQTLs from Braineac database.

geneSymbol	marker	rsid	exprID	chr	start	stop	aveALL	CRBL	FCTX	HIPP	MEDU	OCTX	PUTM	SNIG	TCTX	THAL	WHMT
IKBKE,C1orf147	chr1:206946407	rs1800872	2376833	chr1	206643758	206671379	8.00E-01	5.70E-01	7.90E-01	7.20E-02	3.90E-01	4.10E-01	9.90E-01	**1.50E-04**	9.00E-01	5.80E-02	9.30E-01
FAIM3	chr1:206946407	rs1800872	2452987	chr1	207076630	207101034	**1.30E-02**	3.60E-01	2.30E-01	2.60E-01	5.70E-02	5.60E-01	6.80E-01	2.70E-01	5.20E-01	5.50E-01	**8.40E-04**
LGTN	chr1:206946407	rs1800872	2452902	chr1	206744640	206785896	5.40E-01	7.80E-01	2.30E-01	6.50E-01	8.70E-01	4.50E-01	4.50E-01	**1.20E-03**	6.60E-01	9.90E-01	**1.40E-02**
C4BPA	chr1:206946407	rs1800872	2377185	chr1	207277590	207332055	4.40E-01	**1.20E-03**	9.50E-01	6.40E-01	4.60E-01	3.10E-01	3.30E-01	8.50E-01	9.10E-01	7.20E-01	4.20E-01
CD46,CR1L	chr1:206946407	rs1800872	2377507	chr1	207818478	207983220	1.50E-01	7.40E-01	5.70E-01	2.40E-01	8.00E-01	2.40E-01	7.50E-02	1.80E-01	5.10E-01	**1.60E-03**	4.30E-01
CR1,CR1L	chr1:206946407	rs1800872	2377424	chr1	207669246	207815110	3.00E-01	3.30E-01	1.30E-01	4.50E-01	**2.20E-03**	7.10E-01	2.30E-01	6.00E-02	2.30E-01	4.50E-01	8.30E-01
PIGR	chr1:206946407	rs1800872	2453018	chr1	207101875	207119807	2.90E-01	9.70E-01	8.90E-01	3.50E-01	**2.20E-03**	2.40E-01	7.90E-02	2.50E-01	2.40E-01	6.90E-01	1.40E-01
PIGR	chr1:206946407	rs1800872	2453011	chr1	207101875	207119807	1.80E-01	8.80E-01	9.80E-01	**2.50E-02**	3.60E-01	6.00E-01	4.80E-01	**2.50E-03**	9.60E-01	4.60E-01	**7.00E-03**
IL20	chr1:206946407	rs1800872	2377026	chr1	207038709	207042565	**4.20E-03**	3.20E-01	**2.80E-03**	8.70E-02	6.00E-01	1.10E-01	1.80E-01	3.00E-01	5.00E-01	7.20E-01	1.00E-01
IL10	chr1:206946407	rs1800872	2452956	chr1	206940955	206958002	2.50E-01	**3.50E-03**	**4.00E-02**	6.20E-01	2.20E-01	5.80E-01	**4.00E-02**	9.30E-01	4.00E-01	9.10E-01	6.00E-01

Abbreviations: Ave All, average across all ten regions; CRBL, cerebellum; FCTX, frontal cortex; HIPP, hippocampus; MEDU, medulla; OCTX, occipital cortex; PUTM, putamen; SNIG, substantia nigra; TCTX, temporal cortex; THAL, thalamus; WHMT, white matter. The bold text represents statistically significant results.
